# *GhVTC1*, the Key Gene for Ascorbate Biosynthesis in *Gossypium hirsutum*, Involves in Cell Elongation under Control of Ethylene

**DOI:** 10.3390/cells8091039

**Published:** 2019-09-05

**Authors:** Wangyang Song, Fei Wang, Lihua Chen, Rendi Ma, Xiaoyu Zuo, Aiping Cao, Shuangquan Xie, Xifeng Chen, Xiang Jin, Hongbin Li

**Affiliations:** 1 Key Laboratory of Xinjiang Phytomedicine Resource and Utilization of Ministry of Education, College of Life Sciences, Shihezi University, Shihezi 832003, China; 2 Ministry of Education Key Laboratory for Ecology of Tropical Islands, College of Life Sciences, Hainan Normal University, Haikou 571158, China

**Keywords:** *Gossypium hirsutum*, VTC1, ascorbate biosynthesis, cell elongation, promoter, ethylene

## Abstract

L-Ascorbate (Asc) plays important roles in cell growth and plant development, and its de novo biosynthesis was catalyzed by the first rate-limiting enzyme VTC1. However, the function and regulatory mechanism of *VTC1* involved in cell development is obscure in *Gossypium hirsutum*. Herein, the Asc content and AsA/DHA ratio were accumulated and closely linked with fiber development. The *GhVTC1* encoded a typical VTC1 protein with functional conserved domains and expressed preferentially during fiber fast elongation stages. Functional complementary analysis of *GhVTC1* in the loss-of-function *Arabidopsis vtc1-1* mutants indicated that *GhVTC*1 is genetically functional to rescue the defects of mutants to normal or wild type (WT). The significant shortened primary root in *vtc1-1* mutants was promoted to the regular length of WT by the ectopic expression of *GhVTC1* in the mutants. Additionally, *GhVTC1* expression was induced by ethylene precursor 1-aminocyclopropane-1-carboxylic acid (ACC), and the *GhVTC1* promoter showed high activity and included two ethylene-responsive elements (ERE). Moreover, the 5′-truncted promoters containing the ERE exhibited increased activity by ACC treatment. Our results firstly report the cotton *GhVTC1* function in promoting cell elongation at the cellular level, and serve as a foundation for further understanding the regulatory mechanism of Asc-mediated cell growth via the ethylene signaling pathway.

## 1. Introduction

L-Ascorbic acid (Ascorbate, Asc), also known as vitamin C (VTC), is an essential compound for both animals and plants as the widely existed reductive small molecules in cells, and plays important roles in cell metabolism and physiological response [1,2,3,4]. In plants, Asc performs vital roles in lots of cellular processes such as cell expansion, cell division, and cell wall growth [5,6,7,8]. It also represents one of the most important antioxidants to maintain the cellular reduction/oxidation (redox) homeostasis by detoxifying the cellular excessive reactive oxygen species (ROS) [9] that are commonly generated in association with plant development, cell growth, and the response to environmental stress [10]. As the cofactor of numbers of enzymes such as 1-aminocyclopropane-1-carboxylic acid oxidase (ACO), 2-oxoacid-dependent dioxygenases, violaxanthin deepoxidase, and prolyl-hydroxylase, to keep the metal ion activity in the active center, Asc modulates the plant development, physiological response, and cell growth through the involvement of hormone signaling pathways of ethylene, ABA, and GA [11,12,13,14,15]. Asc and Asc-mediated oxidative molecules indicate significant roles in cell elongation through controlling the redox balance and participating in cell signaling pathways [8,16]. 

Asc concentration varies significantly in different tissues of plant species, ranging from about 20 mM to 300 mM in chloroplasts, showing that a relative dynamic balance of Asc content was maintained to a steady state, which thus decides its crucial physiological functions in cells [17,18]. Asc biosynthesis can occur in all plant tissues. Since the Asc biosynthesis was reported from D-glucose in the hexuronic acid pathway in rats [19], and in higher plants [20,21,22], the biosynthesis of Asc has been properly elucidated with four proposed pathways, including the D-glucose-based Smirnoff–Wheeler (SW) pathway (L-galactose pathway), *myo*-inositol (MI)-dependent D-glucuronate pathway, the D-galacturonic acid pathway deriving from cell wall pectins, and the recycling pathway by the Foyer–Halliwell–Asada cycle [19,23,24,25]. Numerous enzymes locating in these different biosynthesis pathways that indicate the significant determination of the cellular Asc content have been identified and characterized [26,27,28]. In these four pathways, the SW pathway through which the Asc is synthesized from D-glucose has been recognized and fully documented as the predominant pathway in higher plants [20,29,30], which is also proved by in vivo precursor supplement experiments [31], and by genetic evidence of *Arabidopsis* Asc-deficient (*vtc*) mutants [32,33]. All the genes in the SW pathway, including nine continuous reactions from the initiative substrate of D-glucose-6-P to the final product of Asc that is catalyzed by the corresponding enzymes, have been identified clearly [4]. 

The SW pathway is also called L-galactose pathway because of the involvement of the formation of guanosine diphosphate (GDP)-L-galatose (GDP-Gal) converted from GDP-mannose (GDP-Man) [26]. GDP-man is the major precursor for Asc biosynthesis in the SW pathway, and is catalyzed by GDP-mannose pyrophosphorylase (GMP) encoded by the *VTC1* gene. Since the study was firstly reported that the Asc level was promoted to a sevenfold increase in transgenic lettuce and tobacco plants overexpressing the Asc biosynthesis gene of *L-gulono-1,4-lactone oxidase* of rat [34], *VTC1* is considered to be the first key gene to control the Asc biosynthesis [35,36,37,38,39], despite the downstream genes of *GDP-mannose-5′,5′-epimerase* (*GME*), *GDP-galactose phosphorylase* (*GGP*), and *L-galactose-1-phosphate phosphatase* (*GPP*) having been identified as functional regulators to increase Asc content [40,41,42,43,44,45].

Knock-out or knock-down of *VTC1* expression, resulting in the *vtc Arabidopsis* mutants and transgenic plants, supported again that *VTC1* is the crucial gene in the SW pathway to modulate Acs biosynthesis [35,36,46,47]. As the key gene to synthesize cellular Asc, *VTC1* has been identified and characterized in *Arabidopsis* [32,46,47,48], rice [49], kiwifruit [40], potato [35], peach [31], tomato [50,51], and acerola [52]. VTC1 plays diverse important roles in plant development, physiological processes, and response to environmental stress. *VTC1* knock-out *Arabisposis* mutants displayed a significant inhibition of root growth and increased sensitivity to NH_4_^+^ [53], promoted sensitivity to salt stress [54], slow growth and late flowering [36], and delayed senescence and enhanced basal resistance [55]. The transgenic tobacco plants overexpressing or suppressing the tomato *VTC1* gene expression indicated increased or decreased tolerance to temperature stress [50,56]. Antisense downregulation of *VTC1* in potato decreased the Asc content significantly and increased the appearance of dark pots on leaf veins and stems [35]. *VTC1* transcript expression was significantly suppressed in the dark, showing its involvement of light regulation that was associated with the photosynthetic electron transport of the chloroplasts [57]. The transgenic plants by knocking down the expression of the *OsVTC1-1* demonstrated the significant decrease of Asc content, the limitation of light-induced Asc production, and inhibited tolerance to salt stress [49,58]. Radish *VTC1* controlled the taproot development by promoting the Asc accumulation [59].

Upland cotton (*Gossypium hirsutum*) provides the majority of fibers as the most important raw materials for the textile industry, and the fiber cells are the ideal materials to study the cell elongation development that determines the final fiber quality [16,60]. Regarding the Asc metabolism’s important roles in cotton fiber development, our previous studies have indicated that ascorbate peroxidase (APX) showed the ethylene-induced expression that is involved in fiber cell fast elongation development by modulating the ROS homeostasis [16,59,61]; ascorbate oxidase (AO) promoted tobacco cell elongation through generating the apoplast oxidation [8]; *myo*-inositol-1-phosphate synthase (MIPS), which locates in the MI-dependent D-glucuronate pathway, is the key enzyme to synthesize the MI as a precursor to participate in Asc biosynthesis, and is involved in cell elongation [62]. However, to date, the study of the functions of *VTC1* and its controlled Asc biosynthesis in cell elongation and the corresponding regulatory mechanism in *G. hirsutum* has been far from elucidated. In this study, the *G. hirsutum* Asc biosynthesis gene *GhVTC1* was obtained, and indicated the preferential expression during fiber fast elongation stages. Ectopic expression of *GhVTC1* in *Arabidopsis vtc1-1* mutants displayed that *GhVTC1* is a genetically functional gene to rescue the defects of inhibited root growth and retarded development of mutants, and to promote cell elongation of the significant shortened primary root cells of the *Arabidopsis vtc1-1* mutant to the normal length of WT. The isolated 1901-bp promoter region of *GhVTC1* (*PGhVTC1*) was a functional sequence that included two ethylene-responsive-elements (ERE), and the 5′-truncted promoters containing the ERE showed significant increased activity after the ethylene precursor 1-aminocyclopropane-1-carboxylic acid (ACC) treatment. These results indicated that the *G. hirsutum GhVTC1* was a positive regulator to promote the root cell elongation of *Arabipdopsis vtc1-1* mutants under the control of ethylene. 

## 2. Materials and Methods

### 2.1. Plant Materials

*Arabidopsis thaliana* (Col-0) T-DNA insertion mutant *vtc1-1* (stock number: CS8326) was obtained from Arabidopsis Biological Resource Center (ABRC, Columbus, OH, USA). Cotton (*G. hirsutum* L. cv. Xuzhou 142) plants were cultivated in the greenhouse. Different tissues were taken from 2-month-old cotton plants. The day of flowering was defined as 0 days post-anthesis (DPA), and the fibers of different development stages were harvested with detachment from the ovule surface. 

### 2.2. Sequence Alignment and Phylogenetic Analysis of GhVTC1

The *GhVTC1* (Gohir.D10G235400) sequence was obtained by searching the *G. hirsutum* genome in the Joint Genome Institute (JGI) database downloaded from Phytozome (version 12.1.6) via the website of https://phytozome.jgi.doe.gov. Multiple sequence alignment was performed using ClustalW [63], and the conserved domain was analyzed by Pfam [64]. The phylogenetic tree was constructed by MEGA 6 [65] using the neighbor-joining method with bootstrap tests of 1000. 

### 2.3. RNA Extraction and Quantitative Real-Time Polymerase Chain Reaction (qRT-PCR) Analysis 

Total RNA was extracted from 100 mg of frozen materials using an RNAprep Pure Plant Kit (Tiangen, Beijing, China), and the cDNA was synthesized using 1 μg of total RNA from a TIANScript RT Kit (Tiangen, Beijing, China). Agarose gel electrophoresis and a NanoDrop Onec Spectrophotometer (Thermo, Waltham, MA, USA) were used to ensure a high-quality RNA for further utilization (Figure S1, Table S1). All primers were synthesized in Sangon Biotech (Shanghai, China), and double-distilled water (ddH_2_O) was added into the primer dry powder to the concentration of 100 µmol. qRT-PCR was performed using the SYBR Premix Ex Taq (Takara, Kusatsu, Japan) and the designed specific primers (Table S2) on the LightCycler 480 II System (Roche, Basel, Switzerland). A total of 20 µL of reaction mixture was arranged, including 10 µL of SYBR^®^ Premix Ex TaqTM (Tli RNaseH Plus) (2×), 2 µL of cDNA, 0.4 µL of forward primer (10 µM), 0.4 µL of reverse primer (10 µM), and 7.2 µL of ddH_2_O. To detect amplification efficiency, the standard curve was obtained by diluting the cDNA template (Figure S2a), and the amplification efficiency was calculated by the formula E = 10^(−1/slope)^ − 1. The 1% agarose gel electrophoretogram was used to analyze the fragment size of the amplification product, and the melting curves were generated by the LightCycler 480 II System (Figure S2b,c) software to guarantee the accuracy and reliability of the qRT-PCR reactions. The relative expression levels of the target genes were calculated through the 2^−∆∆Ct^ method [66] using the *G. hirsutum ubiquitin 7* (*GhUBQ7)* gene as internal control. 

### 2.4. Vector Construction and Genetic Transformation 

The full-length cDNA of *GhVTC1* coding sequence (CDS) was amplified using the specific primers containing the restriction enzymatic sites of *Kpn* I and *Spe* I. The PCR products and the modified vector of *pCAMBIA2300-GFP* were utilized to generate the *35S::GhVTC1-GFP* vector that was subsequently transformed into *Arabidopsis vtc1-1* mutants with the *Agrobacterium*-mediated floral-dip method [67].

### 2.5. Promoter Activity Analysis of GhVTC1 Promoter 

The 1901-bp sequence upstream of the *GhVTC1* translational start site was obtained from the genome database, and then validated by sequencing. Putative *cis*-acting elements of the *GhVTC1* promoter (*PGhVTC1*) were analyzed by the online software of PLANTCARE at the website of http://bioinformatics.psb.ugent.be/webtools/plantcare/html/ [68]. According to the distribution of the ethylene-responsive element in the promoter region of *GhVTC1*, the 5′-truntcted vectors, including the different *GhVTC1* promoter regions that contained the ERE or not, were constructed into the modified vector of *pCAMBIA1304-GFP-GUS* (Clontech, Mountain View, CA, USA) by replacing the *cauliflower mosaic virus* (*CaMV*) *35S* promoter. The generated series of *PGhVTC1::GFP-GUS* vectors were named P-1901 (–1901/-1, 1901 bp, containing two ERE elements), P-1600 (–1600/-1, 1600 bp, including one ERE element), P-1360 (–1360/-1, 1360 bp, without any ERE elements), P-640 (–640/-1, 640 bp without any ERE elements), and P-240 (–240/-1, 240 bp without any ERE elements). Then, the different 5′-truncted vectors were transformed into tobacco leaves by the transient transformation method for further promoter activity analysis [69]. 

### 2.6. Determination of Asc Content

The Asc content was measured with our previous described method [8]. A total of 0.5 g of materials were ground fully in 2 mL of 6% TCA with successive 7104× *g* centrifugation at 4 °C for 15 min. The supernatant was collected and used for the determination of reduced ascorbic acid (AsA). The reaction mixture included 0.2 mL of extracts, 0.6 mL of 0.2 mol/L PBS (pH 7.4), 0.2 mL of ddH_2_O, 1 mL of 6% TCA, 0.8 mL of 42% H_3_PO_4_, 0.8 mL of 4% 2,2’-dipyridine, and 0.4 mL of 3% FeCl_3_. For the determination of total Asc, including the reduced form of AsA and the oxidized form of dehydroascorbic acid (DHA), 0.2 mL of 0.2 mol/L of DL-dithiothreitol (DTT) was added into the reaction mixture as an external supplement. The reaction solution was heated at 42 °C for 1 h, and then was detected at 525 nm. The Asc standard curve with known concentrations was used to calculate the specific Asc content. The content of DHA was the difference between the total Asc content and the reduced AsA. 

### 2.7. GMPase Activity Measurement

About 0.5 g of *Arabidopsis* leaves were ground to powder in 4 mL of extracted buffer, including 100 mM of Tris pH 7.6, 1% polyvinylpyrrolidone (PVP), 5 mM of DTT, and 1 mM of ethylene diamine tetraacetic acid (EDTA). Then, the homogenate was centrifuged with 16,099× *g* at 4 °C for 10 min, and the supernatant was collected for enzyme activity measurement. The reaction solution contained the components of 50 mM of triethylamine-HCl (pH 7.0), 0.1 mM of EDTA, 2.5 mM of MgCl_2_, 0.1% bovine serum albumin (BSA), 0.5 mM of NADP^+^, 1 mM of β-mercaptoethanol, 1 mM of glucose, 1 mM of adenosine diphosphate (ADP), 1 mM of sodium pyrophosphate, 0.5 U/mL of nucleoside diphosphate kinase, 1 U/mL of hexokinase, and 2 U/mL of 6-p-glucose dehydrogenase. The absorbance at 340 nm was recorded after the addition of 100 μL of enzyme extract into the reaction mixture for 3 min.

### 2.8. Observation of Confocal Laser Microscope

The *Arabidopsis* roots were stained with 5 g/mL of propidium iodide (PI) for 30 min, and then rinsed with sterile water for 1 h. Then, the treated roots were placed on a slide for further cell morphology observation. The prepared *Arabidopsis* roots and the transgenic tobacco leaves were observed using the confocal laser-scanning microscope (Zeiss LSM510, Oberkochen, Germany) to detect the signals with the activation wavelength of 488 nm and collection in 495–550 nm. 

### 2.9. GUS Histochemical Staining Analysis and GUS Activity Determination 

Analyses of β-glucuronidase (GUS) histochemical staining and GUS enzymatic activity were detected as per our previous described method [69]. The tobacco leaves transformed with the 5′-truncted vectors containing diverse *PGhVTC1* regions were immersed in GUS staining solution and incubated at 37 °C in dark culture overnight. The stained tobacco leaves were successively put into 50% and 70% ethanol, and anhydrous ethanol gradually for 1 to 2 h respectively for gradient decolorization, until the negative control was white without any blue remaining. The images were photographed using a microscope (Olympus, Tokyo, Japan). GUS enzymatic activity was measured through the fluorescence-based analysis using 4-methylumbelliferyl-b-glucuronide (4-MUG) as the substrate. The extracted enzyme solution from the transgenic tobacco leaves was quantified by the Bradford protein assay. The reaction was activated by adding the enzyme extract into the GUS assay buffer (50 mM of sodium phosphate buffer pH 7.0, 10 mM of β-mercaptoethanol, 10 mM of Na_2_EDTA, 0.1% sodium lauroyl sarcosine, and 0.1% Triton X-100) containing the substrate of 2 mM of 4-MUG with successive incubation at 37 °C. The reaction was terminated after the addition of the stop buffer (0.2 M of Na_2_CO_3_), and the solution mixture was detected for GUS activity analysis by monitoring the production of 4-methylumbelliferone fluorochrome (4-MU) using the fluorescence spectrophotometer with the excitation wavelength of 365 nm and the emission wavelength of 455 nm. The GUS activity was defined as pmol 4-MU per mg protein per min. 

### 2.10. In Vitro Ovule Culture and Treatment with Exogenous Ethylene Precursor 1-Aminocyclopropane-1-Carboxylic Acid 

In vitro ovule culture was performed with our previous report as described [16]. Cotton bolls were collected at 0 DPA and sterilized in 70% ethanol for 1 min, and then in 0.1% HgCl_2_ for 15–20 min. The bolls were washed by ddH_2_O for 3–5 times after each step of the treatment. The ovules were peeled out from the bolls carefully, and then were placed on the surface of the liquid culture medium for incubation at 30 °C under dark without agitation. After 7-d culture, the ovules were treated by 10 μmol of the ethylene precursor ACC. 

### 2.11. Statistical Analysis

The data statistics analysis was performed with one-way analysis of variance (ANOVA) followed by Bonferroni test using SigmaStat software (Version 4.0) (Systat Software, San Jose, CA, USA). ** and *** represent *p* < 0.01 and 0.001 respectively.

## 3. Results

### 3.1. Determination of Asc Content during Cotton Fiber Development

**Figure 1:**
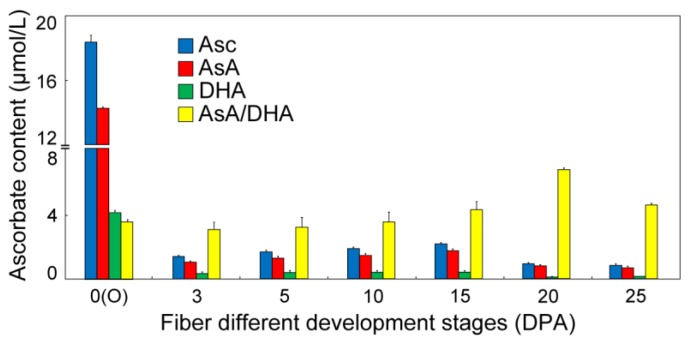
Determination of L-ascorbate (Asc) content and ascorbic acid/dehydroascorbic acid (AsA/DHA) ratio during the fiber development stages. The materials at 0 days post-anthesis (DPA) ovules and the presented ovules’ associated fibers at different development stages were collected for the measurement of Asc content and calculation of the AsA/DHA ratio. Error bars represent the standard error from three independent experiments.

The concentration of Asc was measured during different fiber development stages, showing the results that the total Asc content, the reduced form of AsA, and the oxidized form of DHA were significantly accumulated at 0 days post-anthesis (DPA) ovules (no fiber appearance), and then declined at 3 DPA fibers (Figure 1). During the different points of fiber growth, the content of Asc, AsA, and DHA indicated a similar parabolic curve tendency respectively, reaching the peak value at 15 DPA that is the fiber fast elongation stage. The AsA/DHA ratio maintained a steady status at the stages of 0 to 10 DPA, and showed a significant increase at the stages of 15 to 25 DPA, which are the overlapping time points of fiber fast elongation and secondary wall deposition (Figure 1), suggesting that the redox alteration of the AsA/DHA ratio may perform diverse regulation functions in different fiber development stages. These results indicated that the Asc content and the AsA/DHA ratio were enriched in different fiber development stages, implying their different critical functions. 

### 3.2. Identification and Functional Domain Analysis of Cotton GhVTC1

**Figure 2:**
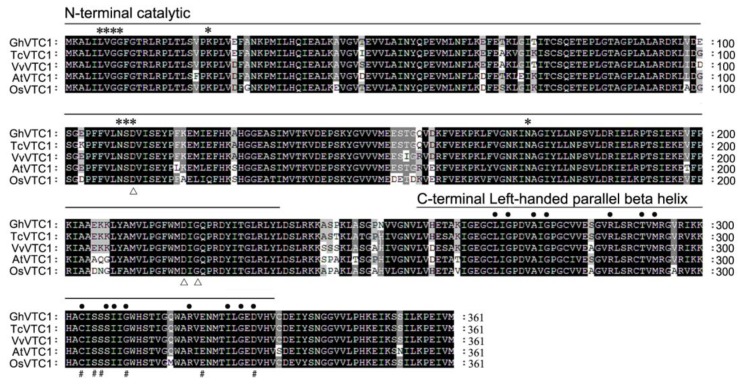
Amino acid sequence alignment and conserved domain analysis of *Gossypium hirsutum* GhVTC1 protein. The protein sequences used for alignment were obtained from Genbank or the Joint Genome Institute (JGI) database with the accession numbers as follows: GhVTC1, Gohir.D10G235400; TcVTC1, XP_007009211; VvVTC1, XP_002282422; AtVTC1, NP_001189713; OsVTC1, XP_015632712. The typical functional domains of the N-terminal catalytic domain and C-terminal left-handed parallel beta helix (LbH) domain are shown. The symbols indicate different functional sites of GhVTC1 with asterisks (*) for substrate-binding sites, hollow triangles (Δ) for metal-binding sites, solid dots (•) for trimer interface sites, and pounds (#) for CoA binding sites.

The full-length *GhVTC1* cDNA was isolated by reverse transcription polymerase chain reaction (RT-PCR) using the RNA samples extracted from fast elongating fibers, with a 1086-bp open reading frame (ORF) and an encoded putative protein of 361 amino acid residues and a predicted molecular weight (MW) of 39.6 kDa. As shown in Figure 2, in alignments with homologous sequences from plant species including *Theobroma cacao*, *Vitis vinifera*, *Oryza sativa*, and *Arabidopsis thaliana*, the GhVTC1 protein belonged to the glycosyltransferase superfamily A (GT-A), and was conservative GDP-mannose pyrophosphorylase, which contains the typical functional domains, including the N-terminal catalytic domain and C-terminal left-handed parallel beta helix (LbH) domain. Many putative functional sites were discovered, including nine substrate-binding sites (asterisks) and three metal binding sites (hollow triangles) both distributing on the N-terminal catalytic domain, and 15 trimer interface sites (solid dots) and six CoA binding sites (pounds) both locating on the C-terminal LbH domain, respectively. These data indicate that GhVTC1 is a conserved enzyme and may perform its function based on these domains. A phylogenetic tree was constructed using the neighbor-joining (NJ) method on the basis of the homologous plant VTC proteins, showing that the VTC proteins were divided into different groups, and that the GhVTC1 was a conserved protein with high similarity with the plant VTC1 homologs (Figure S3).

### 3.3. GhVTC1 is Significantly Accumulated during Fiber Fast Elongation Stages

**Figure 3:**
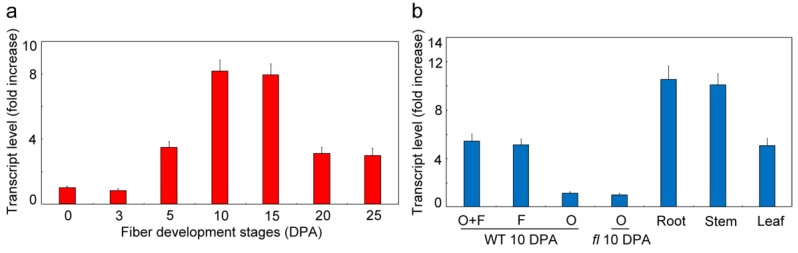
Transcript expression analysis of *GhVTC1* during the fiber development stages. (**a**) *GhVTC1* is preferentially expressed in fast elongating fibers. The wild-type (WT) materials of 0 DPA ovules and presented 3–25 DPA ovules associated with fibers were collected for the detection of qRT-PCR analysis. The qRT-PCR data of 0 DPA ovules was artificially set to 1. (**b**) Tissue-specific expression analysis of *GhVTC1*. The presented materials at 10 DPA, including WT ovules (O), fibers (F), and ovules-associated fibers (O + F), and *fuzzless-lintless* mutant ovules, roots, stems, and leaves were collected for qRT-PCR detection. The qRT-PCR data of 10-DPA *fl* mutant ovules was artificially set to 1. The cotton *ubiquitin* gene *GhUBQ7* (Genbank accession no. XM_016855110) was used as the internal control. Error bars indicate the standard error from three independent experiments.

To analyze the expression pattern of *GhVTC1* as a basis to study its potential function, qRT-PCR was used to determine its transcript expression level in different fiber developmental stages. *GhVTC1* was abundantly expressed from 5 to 25 DPA of the fiber development stages, which includes the fiber fast elongation process, and reached the peak value of ovules-associated fibers at 10 DPA (Figure 3a). Meanwhile, the tissue-specific expression analysis was also performed, indicating that *GhVTC1* was mainly accumulated in 10 DPA fibers but maintained a low value in 10 DPA WT ovules or *fuzzless-lintless* (*fl*) mutant ovules. The latter do not contain any fibers, and are commonly used as control to investigate fiber development, despite the preferential enrichment in roots, stems, and leaves (Figure 3b). The results indicate that *GhVTC1* is a fiber-specific gene and presents preferential accumulation in the fiber fast elongation process, suggesting its potential important function in cell elongation development. 

### 3.4. Functional Complementary Analysis of GhVTC1 in the Loss-of-Function Arabidopsis vtc1-1 Mutants 

**Figure 4:**
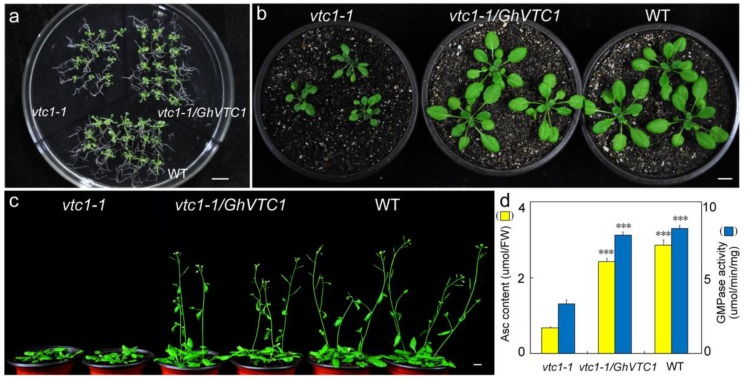
Functional complementary analysis of *GhVTC1* in the loss-of-function *Arabidopsis vtc1-1* mutants. The phenotypes of *Arabidopsis* plants including *vtc1-1* mutants, WT, and transgenic *vtc1-1/GhVTC1* by the ectopic expression of *GhVTC1* in *vtc1-1* mutants were observed. The 2-week-old seedlings (**a**), 4-week-old seedlings (**b**), and 6-week-old plants (**c**) were photographed for phenotype analysis. Bars = 1 centimeter (CM). (**d**) The Asc content and GMPase activity were measured in *vtc 1-1*, *vtc1-1/GhVTC1*, and WT *Arabidopsis* materials. ***, *p* < 0.001 compared to *vtc1-1*. Error bars display the standard error from three independent experiments.

Since the Asc-deficient mutant *vtc1* was isolated, it has long been generally utilized to study the functions in Asc biosynthesis and plant development [35,36,46,47,48], and in light of the significant enrichment of *GhVTC1* in fiber cell elongation stages (Figure 3); thus, *GhVTC1* and the *pCAMBIA2300* vector were used to construct the overexpression vector *35S::GhVTC1*, which was then transformed into the loss-of-function *Arabidopsis vtc1-1* mutants, to investigate the genetic function of *GhVTC1* in vivo. The *vtc1-1* mutants displayed obvious suppressed development with the growth inhibition of roots, leaves, and whole plants, and late flowering (Figure 4a–c), coupled with the significant decrease of both Asc content and the VTC1 enzyme activity (Figure 4d). The transgenic *vtc1-1/GhVTC1 Arabisopsis* plant lines obtained by ectopic expressing cotton *GhVTC1* to the *vtc1-1* mutants indicated a normal phenotype with WT, as well as the recovery of Asc concentration and the VTC1 enzyme activity. These data suggest that *GhVTC1* is a functional gene to rescue the abnormal phenotype of *vtc1-1* mutants by complementing the deleted expression of *Arabidopsis AtVTC1*. 

### 3.5. GhVTC1 Promotes the Root Cell Elongation of Arabidopsis vtc1-1 Mutants

**Figure 5:**
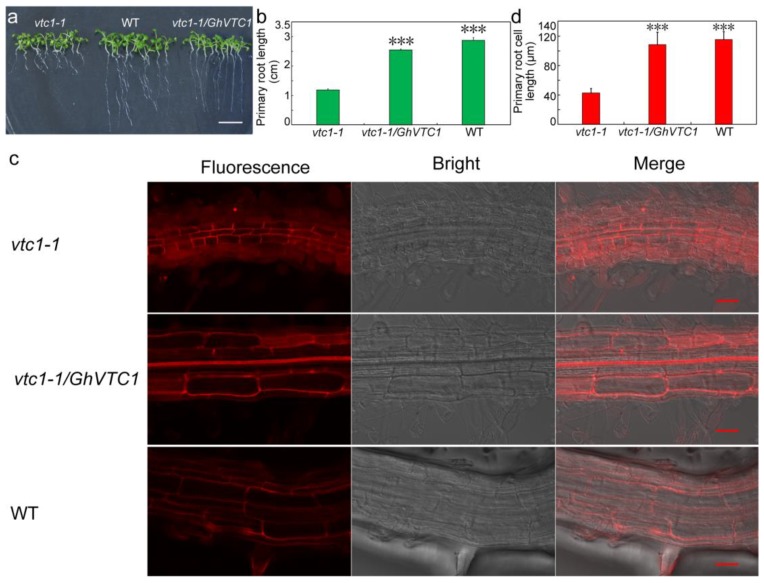
*GhVTC1* promotes the root cell elongation of *Arabidopsis vtc1-1* mutants. The phenotypes of primary roots of *vtc1-1*, *vtc1-1/GhVTC1*, and WT *Arabidopsis* materials were observed (**a**), and the primary root lengths were measured (*n* = 30) (**b**). (**c**) Fluorescence images of *Arabidopsis* primary root cells by confocal laser-scanning microscopy. (**d**) The root cell lengths were measured (*n* = 100). Bars = 1 CM (in **a**) and 50 μm (in **d**). ***, *p* < 0.001 compared to *vtc1-1*.

The important functions of *VTC1* and its catalyzed Asc production in root development [36], and the abundant expression of *GhVTC1* in fast elongating fibers (Figure 3), indicate the potential important role of *GhVTC1* for plant cell elongation. Thus, we further determined the length of the primary roots and root cells in *Arabidopsis* plants. The primary roots of *vtc1-1* mutants were significantly shortened with the length of 1.12 ± 0.037 CM, regarding the normal root lengths in the *vtc1-1/GhVTC1* transgenic *Arabidopsis* lines and WT with the lengths of 2.56 ± 0.035 and 2.9 ± 0.084 CM, respectively (Figure 5a,b), suggesting the important function of *GhVTC1* in promoting the cell elongation of root cells of *Arabidopsis vtc1-1* mutants to be normal. Further confocal microscopy detection displayed that the primary root cell lengths were 43.23 ± 6.32 μm in *vtc1-1*, 109.56 ± 17.05 μm in *vtc1-1/GhVTC1*, and 116.80 ± 10.67 μm in WT (Figure 5c,d), indicating again the crucial role of *GhVTC1* in root cell elongation at the cellular level. The genetically functional complementary analysis suggests that *GhVTC1* is a fully functional gene to control root development, and performs a key role in cell elongation as a positive regulator. 

### 3.6. GhVTC1 is the Ethylene-Induced Gene by the ERE Element Distributing on the Promoter Region

**Figure 6:**
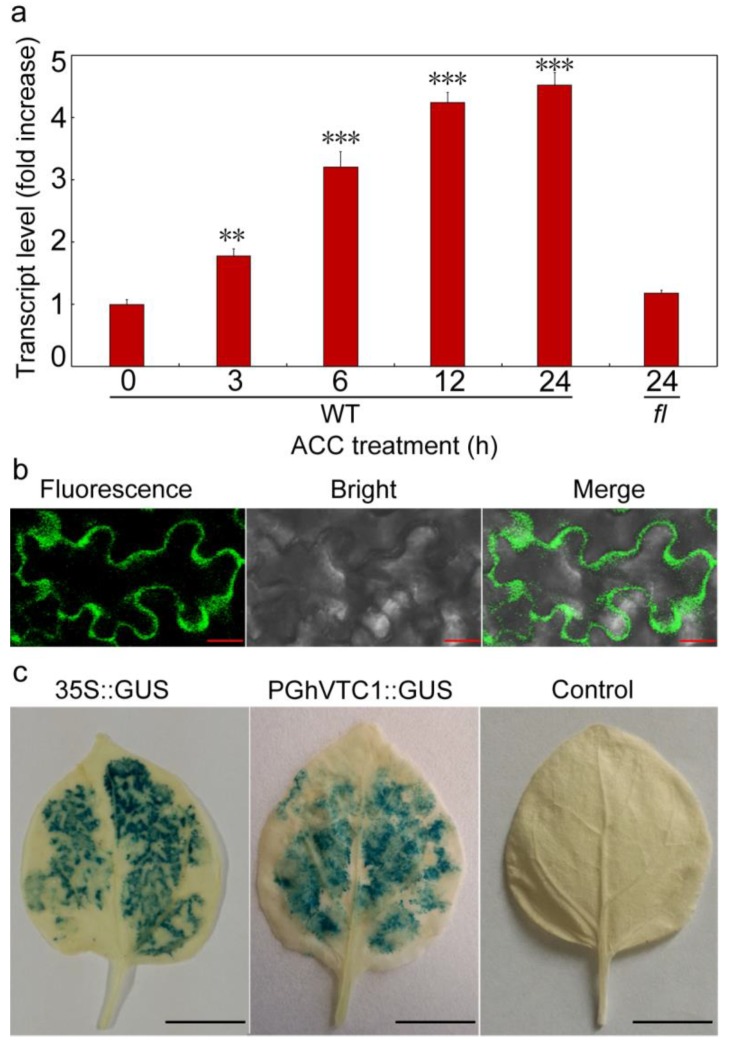
Analyses of transcript expression of *GhVTC1* under aminocyclopropane-1-carboxylic acid (ACC) treatment and promoter activity of *PGhVTC1*. (**a**) Expression analysis of *GhVTC1* under ACC treatment at the transcriptional level. The WT and fuzzless-lintless (*fl*) mutant ovules were cultured for 7 days in vitro, and then were treated by ACC as presented time points. The treated cotton materials were used for RNA extraction and successive qRT-PCR analysis. (**b**) Fluorescence detection of green fluorescent protein (GFP) expression in *PGhVTC1* transgenic tobacco leaves. The *PGhVTC1::GFP-GUS* transgenic tobacco leaves were used to detect the GFP expression (bright field, fluorescence, and merged images) by fluorescence microscopy. (**c**) GUS histochemical staining of transgenic *PGhVTC1::GFP-GUS* tobacco leaves. Bars = 50 μm (in **b**) and 1 CM (in **c**). ** and *** indicate *p* < 0.01 and 0.001 compared to 0-h WT.

**Figure 7:**
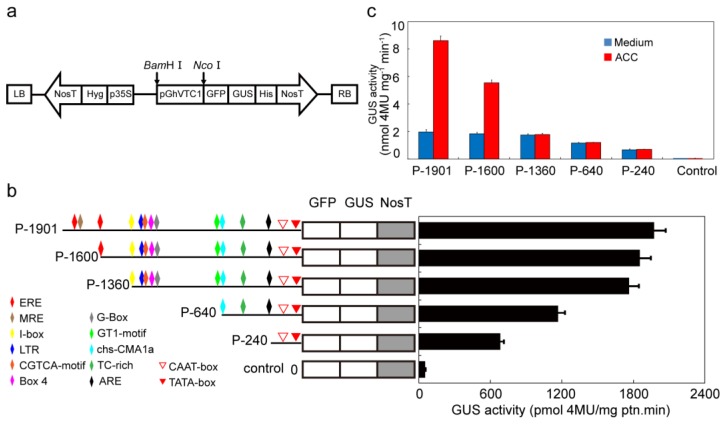
Promoter activity analysis of the 5′-truncted *PGhVTC1*. (**a**) Schematic presentation of the 5′-truncted constructs. The full-length and 5′-truncated fragments of *PGhVTC1* were constructed into the modified *pCAMBIA1304-GFP-GU*S vector using the various 5′-truncted *PGhVTC1* to replace the *CaMV35S* promoter to drive the expression of the *GFP-GUS* gene, generating the constructs P-1901, P-1600, P-1360, P-640, and P-240. The constructed 5′-truncted *PGhVTC1* vectors were transformed into tobacco leaves using the *Agrobacterium*-mediated transient transformation method. (**b**) Quantitative analysis of the GUS activity of different 5′-truncted *PGhVTC1*. The GUS activity was measured in transgenic tobacco leaves transformed with the different 5′-truncted *PGhVTC1* constructs. The GUS activity was defined as pmol 4-MU per mg protein per min. The data are presented as the average of three independent experiments. The colored different symbols represent the *cis*-acting elements distributing on the *GhVTC1* promoter regions at the corresponding sites. (**c**) Promoter activity analysis of 5′-truncted *PGhVTC1* constructs under ACC treatment. The various 5′-truncted *PGhVTC1* transgenic tobacco leaves were treated with or without 10 µmol of ACC. Then, the GUS activity was determined by the fluorescence spectrophotometer. The GUS activity of non-DNA transformants (control) was also investigated. Each value represents the mean of the results from three independent experiments, and the bars indicate standard deviations.

In the light of the essential function of the phytohormone ethylene in the elongation of fiber cells [60,70], as well as the regulation of Asc metabolism-related genes by ethylene [16,61], the transcript expression analysis of *GhVTC1* under ethylene treatment was performed, to investigate the regulation relationship between *GhVTC1* and ethylene. The materials of ovules and fibers that were obtained by in vitro ovule culture with the supplement of the ethylene precursor ACC were used to extract the RNA samples, and then were utilized as templates for qRT-PCR detection. The results indicated that the prompt induced expression of *GhVTC1* appeared significantly after 3-h treatment of ACC (Figure 6a). Additionally, to explore the further regulatory mechanism, the 1901-bp promoter sequence upstream of the translational start site was isolated with reference to the *GhVTC1* cDNA sequence. The *GhVTC1* promoter (*PGhVTC1*) included many putative plant *cis*-elements (Table S3), such as the core elements of TATA-box and CAAT-box, and the regulatory elements of light responsive elements (Box I, ACE, I-box, Box 4, G-box, TCT-motif, GT1-motif, chs-CMA1a and GTGGC-motif), stress responsiveness elements (LTR for low-temperature response, and HSE for heat stress response), and the transcription factor binding element of MRE for MYB binding. Interestingly, some plant hormone responsiveness elements were also discovered, including ethylene-responsive element (ERE: ATTTCAAA), MeJA-responsiveness (CGTCA-motif: CGTCA), and auxin-responsive element (TGA-element: AACGAC). To assess the promoter activity, the *PGhVTC1::GFP-GUS* vector was constructed and introduced into tobacco leaves by the *Agrobacterium*-mediated transient transformation method. The green fluorescence was detected with significant accumulation in the cells of the transgenic tobacco leaves by laser confocal microscope (Figure 6b). Meanwhile, the abundant blue deposition was observed obviously in the transgenic tobacco leaves by GUS histochemical staining analysis (Figure 6c), indicating that the *PGhVTC1* is a functional sequence to effectively activate the expression of the reporter genes *GFP* and *GUS*. Regarding the important role of ethylene (ETH) in fiber cell elongation and the existence of ERE distributing in *PGhVTC1*, to investigate the potential regulation pattern between *PGhVTC1* and ETH, the diverse vectors containing the different 5′-truncted *PGhVTC1* regions were designed and constructed with or without the involvement of ERE, as well as the *cis* elements presented in the different 5′-truncted promoters with different colored symbols (Figure 7a,b). The different 5′-truncted promoters exhibited distinct activity to drive *GUS* expression to generate different GUS enzyme activity, with a decreased tendency gradually along with the reduction of the promoter sequence length (Figure 7b). Moreover, the transgenic tobacco leaves transformed with *PGhVTC1::GFP-GUS* were treated by ACC to study the relationship between *PGhVTC1* expression and ethylene. After feeding of exogenous ACC, the GUS enzyme activity showed a prompt increase in transgenic tobacco leaves transformed with the ERE-containing promoters (P-1901 and P-1600), while maintaining a steady low level in transgenic tobacco leaves transformed with the ERE-deletion promoters (P-1360, P-640, and P-240) (Figure 7c). These data suggest that *GhVTC1* expression is modulated by ethylene through the ERE locating on the promoter region, and may be involved in the ethylene-mediated signaling pathway. 

## 4. Discussion

Asc plays diverse roles in cell growth and plant development, and its de novo biosynthesis has long been demonstrated by the SW pathway, in which VTC1 is the key enzyme to catalyze the conversion of GDP-Man to GDP-gal [26]. The Asc content was significantly accumulated in the fiber initiation stage of 0 DPA ovules, and during the fiber development process from 3 to 25 DPA, the contents of Asc, AsA, and DHA displayed bell-shaped curves with the peak values at 15-DPA fibers, and the AsA/DHA ratio appeared to have a gradual increase, with the highest level at 20-DPA fibers (Figure 1), showing a similar report as that in cotton [71]. It is commonly deemed that the ROS burst is a key factor for fiber initiation [72] and *Arabidopsis* root hair elongation [10,73], and the ROS accumulation shows an important function for fiber secondary deposition [61,71]. Asc is known as the important antioxidant to eliminate ROS to maintain the cellular redox balance [9]; thus, it is easy to understand the significant accumulation of Asc at 0-DPA ovules and at 20-DPA fibers as reductive molecules to detoxify the redundant cellular ROS. 

Lots of *VTC1* genes have been identified in many plant species. The *GhVTC1* was obtained from the developing fibers of *G. hirsutum*, and was the typical member of glycosyltransferase superfamily A with conserved domains and functional sites that endow it to fulfill the GMPase function to catalyze the Asc biosynthesis (Figure 2, Figure S3). The transcription expression of *GhVTC1* indicated specific abundant expression during the fiber fast elongation development process (Figure 3), suggesting the important function of *VTC1* in cell growth. The mutation in GMPase resulted in the function deficiency of *AtVTC1*, and thus inhibited the root growth in *Arabisopsis* [53], and slower shoot growth and suppressed plant development were observed in the *VTC1* function deletion mutant *vtc1-1* [36,55]. The latter indicated that the lower levels of GDP-mannose and the altered protein N-glycosylation were generated, and thus resulted in root growth inhibition in *vtc1-1* mutants [47]; similar reports were also discovered in *Arabidopsis* [74]. GDP-mannose and protein glycosylation are necessary for normal cell wall formation, which is the key factor to determine cell growth [47]. The N-glycosylation deficiency can lead to the inhibition of protein synthesis, which may be mediated by indole-3-acetic acid (IAA), which is essential for cell elongation [75]. The significant inhibition of root development and late flowering in *Arabidopsis vtc1-1* mutants were indicated, which was recovered in the transgenic *Arabidopsis* lines overexpressing the *GhVTC1* in *vtc1-1* mutants (Figure 4 and Figure 5), suggesting the complete important function of *GhVTC1* in plant development, and providing the possible involvement of *GhVTC1* in IAA-mediated protein synthesis. 

The cell wall is the crucial component to decide the final property of the cell, including the length and mechanical strength that control the cell shape morphogenesis [76]. The process of cell elongation or extension is determined by cell wall alterations such as wall softening and reorganization [77]. The Asc biosynthetic pathway is closely correlated with cell wall biosynthesis. The precursors of GDP-Man and GDP-Gal in the SW pathway are the important substrates to provide the glycosyl residues to synthetize pectins and hemicelluloses, which are the major components of the cell wall [28]. VTC1 is the key enzyme to catalyze the formation of GDP-Man [26,46,78], showing its potential function in cell wall biosynthesis and thus the regulation of cell growth. Significant growth inhibition or arrest is observed due to the cell wall formation defect that is caused by the Asc decrease induced by the deficiency or suppression of the related genes in the SW pathway [47,79,80]. The *Arabidopsis AtVTC1* knock-out mutant displayed a significant alteration of cell wall composition that led to the cease of embryo development [47], implying the close regulation link between VTC1 and cell wall formation to control cell growth and plant development. Supporting again the connection between Asc and cell growth affected by cell wall structure, suppression or overexpression of the *Arabidopsis KONJAC* gene that is the key element to activate GMP activity resulted in a significant decrease or increase of the glucomannan content of cell walls [81]. In this study, the cotton *GhVTC1* gene is preferentially accumulated in fast elongating fibers (Figure 3), and the overexpression of *GhVTC1* in the root-cell-shortened *vtc1-1 Arabidopsis* mutants rescued the defects to normal or WT (Figure 4 and Figure 5), indicating that cotton *GhVTC1* performs a key function in cell growth through the possible regulation of cell wall formation. During fiber fast elongation stages, by comparative proteomics study, the pectin biosynthesis proteins that are involved in cell wall formation were characterized to promote the cell elongation of cotton fibers and *Arabidopsis* root hairs [82]. Asc can be used as the enzyme cofactor of proline and lysine hydroxylases that catalyze the production of hydroxyproline-rich glycoproteins (HRGP), including arabinogalactans (AGP) and extensins (EXTs). These proteins are important structural components to stimulate cell elongation or expansion by affecting cell wall assembly, through covalent attachment to pectins and hemicelluloses [83,84,85,86,87]. 

The oxidative generation induced by the oxidative molecules of ROS and DHA that have been demonstrated to play important roles in cell division and expansion is the key factor to control cell growth by regulating the cellular redox homeostasis [73,76,88,89]. Asc is the common substrate to eliminate the excessive ROS of the cell, and can be oxidized to produce the DHA, showing the close link between it and the cellular oxidative molecules as well as the potential function to involve in cell elongation. The members of ROS, H_2_O_2_, and the hydroxyl radical display the involvement of cell growth [10]. Significant H_2_O_2_ accumulation during the fiber fast elongating stages and the promotion of fiber cell elongation by the supplement of exogenous H_2_O_2_ that may be caused by inducing cell wall loosening were observed [16,72,90,91]. DHA has been indicated to participate in cell expansion by the induction of plasma membrane depolarization and the enhancement of cell wall softening [92,93]. A significant accumulation of apoplast oxidation was discovered in cotton fibers and induced the tobacco cell elongation [8]. Apoplast is the key area to decide the signal transduction from cell to cell and from the outside to inside of the cell, and thus affect cell development [76,94]. It has been reported that Asc is implicated with cell wall softening by the enrichment of hydroxyl radical concentration in the apoplast [95]. Asc is the only antioxidant reduced molecule to regulate the cellular redox status by affecting ROS and DHA contents, implying its possible important role in simulating cell growth. 

Ethylene has long been extensively investigated to regulate plant development and physiological response [96,97,98], and acts as a positive regulator to control the root hair and hypocotyl development [99,100,101,102,103]. The deficiency of ethylene signal transduction in *Arabidopsis* mutants resulted in shortened root hairs, despite of the recovery by the supplement of exogenous ethylene precursor ACC [99,104,105]. During the fiber elongation stages, ethylene biosynthesis is one of the most significantly upregulated pathways, and the accumulated expression of ethylene biosynthesis gene *ACOs* in fast elongating fibers and increased fiber elongation by the application of exogenous ethylene were observed, showing the major role of ethylene in promoting cotton fiber elongation [58,60]. A very high expression level of *ACO* is the decisive factor to the ethylene burst that has been demonstrated to be the key modulator for fiber cell growth. Furthermore, the putative MYB-binding site distributing on the promoter region of *ACO* appeared to be the crucial sequence to regulate the *ACO* expression and the subsequent ethylene production. Both the deletion of the MYB-binding site and inactivation or overproduction of *ACO* resulted in the inhibition of fiber growth, suggesting the precise and comprehensive regulation mechanism for *ACO* expression and the ethylene biosynthesis during fiber cell development [106]. Asc is the important cofactor for ACO to maintain the enzyme activity, suggesting its potential connection with the ethylene signaling pathway. The ethylene response factor (ERF) proteins, as one member of the AP2/EREBP transcription factor superfamily [70,107], is the key factor to transduce the ethylene signal in cells, and thus performs important functions in cell growth and plant development [70,108,109]. The promoted Asc content regulated by *Arabidopsis AtERF98* was indicated, with increased and decreased Asc levels in overexpressing lines and knock-out mutants *atref98-1* and *atref98-2*, indicating the controlled Asc biosynthesis by ethylene. Overexpressing *AtERF98* in *vtc1-1* mutants recovered the Asc content, showing that ethylene is the upstream factor to regulate Asc biosynthesis, mainly through the D-Man/L-Gal synthesis pathway [110]. In this study, the expression of cotton *GhVTC1* was significantly induced by ethylene (Figure 6a), and there exists the ERE element in the promoter of *GhVTC1* (Table S3, Figure 7b). The promoters containing the ERE element displayed a significant increase of promoter activity by the supplement of exogenous ethylene precursor ACC (Figure 7c), validating again the possible regulation of Asc biosynthesis by ethylene through affecting the expression of *VTC1*. The interaction of AtERF98 binding to the promoter of *AtVTC1* was indicated by transient expression and chromatin immunoprecipitation assays [110]. Analyzing the further mechanism of ethylene to regulate Asc biosynthesis and the possible interaction between ERF and *GhVTC1* in *G. hirsutum* are warranted. All these suggest that VTC1 and its regulated Asc biosynthesis are involved in cell elongation under the control of ethylene. In spite of the well-elucidated cell signaling of Asc biosynthesis participating in cell elongation, whereas, at the organelle level, the distribution of Asc is diverse in different organelles with different concentrations [111], further studies on the transport of Asc across different compartments of the cell and the corresponding functions are helpful for us to understand the Asc metabolism. 

## 5. Conclusions

We obtained the *G. hirsutum* Asc biosynthesis gene *GhVTC1* that encoded a typical plant VTC1 protein with functional conserved domains and was expressed preferentially in elongating fibers. Further genetically functional complementary analysis by ectopic expressing *GhVTC1* in the *Arabidopsis vtc1-1* mutant showed that *GhVTC1* is functional to rescue the abnormal phenotypes of inhibited root growth and retarded development, and to promote the cell elongation of significant shortened root cells in *vtc1-1* mutants as normal length in WT. Moreover, the activity analysis of 5′-truncted promoters indicated that the promoter regions of *PGhVTC1* containing the ERE had significant increased activity after ethylene precursor ACC treatment. These results suggest that the *G. hirsutum GhVTC1* is a positive regulator to promote root cell elongation, and provides the basis to elucidate the regulatory mechanism of Asc-mediated cell growth in a hormone manner.

## Supplementary Materials

The following are available online at https://www.mdpi.com/2073-4409/8/9/1039/s1. Table S1. Quality check of RNA samples. Table S2. Primers used in this work. Table S3. Putative *cis*-acting elements in the promoter region of *GhVTC1*. Figure S1. Quality check of the RNA samples by electrophoresis detection. Figure S2. Primer detection of *GhVTC1* and *GhUBQ7*. Figure S3. Phylogenetic tree of GhVTC1 and other plant VTC proteins.

## Abbreviations

ACC	1-aminocyclopropane-1-carboxylic acid
ACO	1-aminocyclopropane-1-carboxylic acid oxidase
AO	Ascorbate oxidase
AsA	Ascorbic acid
Asc	Ascorbate
DHA	Dehydroascorbic acid
DPA	Day post-anthesis
ETH	Ethylene
GDP	Guanosine diphosphate
GDP-Gal	GDP-L-Galatose
GDP-Man	GDP-Mannose
GFP	Green fluorescent protein
GGP	GDP-galactose phosphorylase
GME	GDP-mannose-5′,5′-epimerase
GMP	GDP-mannose pyrophosphorylase
GUS	β-glucuronidase
MEGA	Molecular evolutionary genetics analysis
MIPS	*Myo*-inositol-1-phosphate synthase
ROS	Reactive oxygen species
SW	Smirnoff–Wheeler
VTC	Vitamin C
WT	Wild type
