# Supplementary material for: GhVTC1, the Key Gene for Ascorbate Biosynthesis in Gossypium hirsutum, Involves in Cell Elongation under Control of Ethylene
Source: Cells. 2019 Sep 5;8(9):1039. doi: 10.3390/cells8091039 (PMC6769745; doi:10.3390/cells8091039)
Supplement: Supplementary file 1 [file cells-08-01039-s001.zip › Supplementary files_R1_Proof_submit/Supplementary Figures-R1.doc]

**Supplementary Figure S1.** Quality check of the RNA samples by electrophoresis detection. The two distinct bands represent the 28S and 18S RNAs respectively, and the bands of DNA marker are also shown.

**Supplementary Figure S2.** Primer detection of *GhVTC1* and *GhUBQ7*. **a** The amplification efficiency analysis of the primers. The primers of *GhVTC1* and *GhUBQ7* were used to amplify the different dilution concentrations of cDNA templates by qRT-PCR. The horizontal coordinate represents the dilution ratio of cDNA, and the vertical coordinate delegates the CP value obtained from qRT-PCR. **b** The amplification fragment length detection by qRT-PCR. The single band was generated by the amplification of *GhVTC1* or *GhUBQ7* using the corresponding gene-specific primers. Control 1 and Control 2 were references for the amplifications of *GhUBQ7* and *GhVTC1* respectively, using ddH_2_O to replace the cDNA templates. **c** The Melting Curve of qRT-PCR for *GhVTC1* and *GhUBQ7*.

**Supplementary Figure S3.** Phylogenetic tree of GhVTC1 and other plant VTC proteins. The phylogenetic tree was constructed by MEGA6.0 using neighbor-joining method with 1000 bootstrap replicates. The protein sequences were obtained from Genbank and JGI database with the accession numbers as follows: GhVTC1, Gohir.D10G235400; AtVTC1, NP_001189713; AtVTC2, NP_567759; AtVTC5, NP_200323.1; AtVTC3, NP_001325404; OsVTC2, XP_015632712; OsVTC1, XP_015632712; ZmVTC1, NP_001142302; ZmVTC2, NP_001150222. GmVTC1, Glyma18g03840; GmVTC2/5, Glyma08g43200; GmVTC4, Glyma07g39620; GmVTC3, Glyma02g21970; MtVTC1, Medtr5g080770; MtVTC2/5, Medtr3g053020; MtVTC4, Medtr2g026060; MtVTC3, Medtr1g050520.
